# Exploration of the correlation between AHNAK2 and pancreatic cancer and its role in the tumor microenvironment based on bioinformatics: Computational pharmacology

**DOI:** 10.1097/MD.0000000000046827

**Published:** 2025-12-26

**Authors:** Bo Wu, Yixin Liu, Xiaohong Lan, Yuekun Wang, Yang Yang, Xuqing Chen

**Affiliations:** a Department of Pharmacy, Jinling Hospital, Affiliated Hospital of Medical School, Nanjing University, Nanjing, China; b The 8th Posting Clinic of the Eastern Theater General Hospital, Nanjing, China.

**Keywords:** AHNAK2, bioinformatics, PAAD, tumor microenvironment

## Abstract

Pancreatic adenocarcinoma (PAAD) ranks as a leading global cause of cancer-related mortality. Current standard treatments, including surgery, radiotherapy, and chemotherapy, offer limited survival advantages to patients. Immunotherapy, which has achieved remarkable success in prolonging overall survival (OS) in lung cancer patients, has yet to reach its full potential in PAAD. Only a subset of patients responded to immunotherapy, highlighting the urgent need to identify reliable immunotherapy biomarkers for better patient selection. To unearth immune prognosis-associated genes in PAAD, we harnessed R software and the TIMER database to retrieve raw data from The Cancer Genome Atlas and Gene Expression Omnibus. The expression patterns of target genes were comprehensively analyzed across The Cancer Genome Atlas and Gene Expression Omnibus datasets. R software and the TISIDB database were utilized for Gene Ontology (GO) and Kyoto Encyclopedia of Genes and Genomes enrichment analyses. Additionally, correlations with tumor immune profiles were explored. Through this systematic screening, we identified AHNAK Nucleoprotein 2 (AHNAK2) as a promising immune prognosis-related gene. AHNAK2 exhibits significant differential expression between PAAD and normal tissues, positioning it as a valuable biomarker for PAAD diagnosis and prognosis assessment. Enrichment analysis reveals that differential genes related to AHNAK2 are predominantly involved in epidermis development, keratinization, intermediate filament organization, and epithelial cell differentiation. Notably, AHNAK2 expression positively correlates with the infiltration levels of memory B cells (*r* = 0.17, *P* < .05), regulatory T cells (Tregs) (*r* = 0.21, *P < *.001), and macrophages (*r* = 0.35, *P* < .001). Drug susceptibility analysis further demonstrates that AHNAK2 confers significant resistance to bafetinib (*r* = −0.38, *P* < .05) and curcumin (*r* = −0.55, *P* < .05). Our study provides compelling evidence of a robust association between AHNAK2, the prognosis of PAAD, and the tumor immune microenvironment. The findings underscore the potential of AHNAK2 to serve as a valuable biomarker for immunotherapy in PAAD. This discovery paves the way for the development of more tailored and efficacious treatment approaches, offering new prospects for improving patient outcomes and advancing the field of PAAD management.

## 1. Introduction

**Figure 1.**
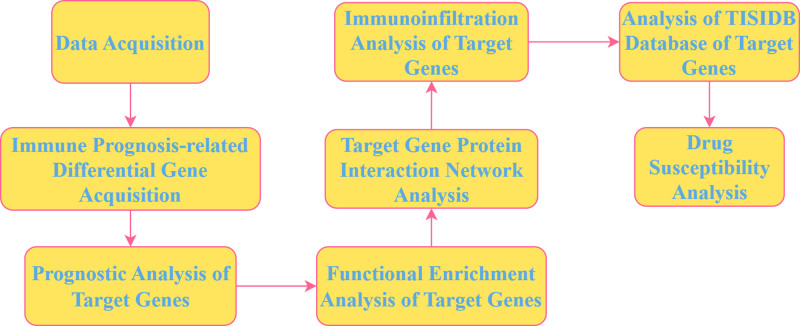
Schematic representation of the study workflow. (Image source: https://app.diagrams.net/).

Pancreatic adenocarcinoma holds the 14th position globally in terms of the number of new cases and ranks 7th regarding new deaths among common malignant tumors.^[1]^ Traditional treatments including surgery, chemotherapy, radiotherapy, and targeted therapy have long been the standard treatment options for pancreatic adenocarcinoma (PAAD). Nevertheless, the survival benefits that PAAD patients can obtain from these conventional treatments are rather limited. Under normal physiological conditions, the immune system plays a pivotal role in anti-tumor activities. It can recognize cancer cells and, upon identifying tumor-associated antigens, initiate appropriate immune responses to eliminate these cells. This mechanism has brought cancer treatment one step closer to the realm of precision medicine.^[2–4]^ The results of several clinical studies^[5–7]^have shown that immunotherapy has better efficacy and fewer side effects than traditional chemotherapy. However, only a small proportion of patients can derive benefits from immunotherapy. Therefore, there is a need to explore more potential biomarkers for immunotherapy to screen out populations that are more likely to respond favorably.^[8,9]^ High-throughput sequencing technology, as an emerging approach for detecting oncological diseases, has achieved significant advancements in genomic variant detection and clinical applications.^[10,11]^ High-throughput sequencing technology enables us to gain a more profound understanding of the pathogenesis of PAAD and facilitates the further screening of biomarkers for the diagnosis, treatment, and prognostic evaluation of PAAD.^[12]^ In the present study, building upon the above, bioinformatics approaches were employed to screen genes associated with the prognosis and immunity of PAAD within the The Cancer Genome Atlas (TCGA) and Gene Expression Omnibus (GEO) datasets. Furthermore, the expression of the target genes in PAAD tissues and their correlations with the PAAD immune microenvironment were investigated, thus providing potential biomolecular markers for PAAD immunotherapy. The technical roadmap for this study is shown in Figure 1.

## 2. Materials and methods

### 2.1. Data acquisition

For the data collection process, publicly available datasets from TCGA and GEO (https://www.ncbi.nlm.nih.gov/geo/) were utilized. In the TCGA dataset, high-quality genomic, transcriptomic, and clinical data related to PAAD patients were retrieved. Rigorous quality control measures were applied to ensure the integrity and reliability of the TCGA data. All the data underwent pre-processing steps such as normalization and batch effect correction to eliminate potential confounding factors.^[13,14]^ Regarding the GEO dataset, 2 gene chips (GSE28735 and GSE62452) were obtained. GSE28735 contains 45 PAAD samples and 45 adjacent non-tumor tissue samples, with data from the GPL6244 platform. GSE62452 contains 69 PAAD samples and 61 adjacent non-tumor tissue samples, also with data from the GPL6244 platform. The relevant gene expression profiles of PAAD samples from these 2 gene chips were carefully selected based on predefined inclusion criteria. These criteria included the sample size, sample source, and experimental methods used in the original studies. Data from distinct GEO series were standardized to ensure comparability. Multiple GEO datasets were amalgamated to enhance the statistical power and comprehensiveness of the analysis. By integrating the extensive information derived from both the TCGA and GEO datasets, a more holistic and precise understanding of PAAD-related genes and their functions could be achieved. This integration laid a robust foundation for subsequent bioinformatics investigations.

The TCGA program, a collaborative initiative between the National Cancer Institute (NCI) and the National Human Genome Research Institute, was initiated in 2006. Currently, it is engaged in the study of a total of 36 distinct cancer types. Employing large-scale sequencing-based genome analysis techniques, TCGA aims to unravel the molecular mechanisms underlying cancer through extensive collaborative efforts. By doing so, it seeks to enhance the scientific understanding of the molecular basis of cancer pathogenesis, with the ultimate goal of improving our capabilities in cancer diagnosis, treatment, and prevention.^[15,16]^ RNA-seq data from the TCGA database (https://portal.gdc.cancer.gov) for the STAR process of the TCGA-PAAD project, along with clinical data, were downloaded and compiled. This dataset encompasses 178 PAAD samples and 4 normal lung tissue samples.

### 2.2. Identification of immune prognosis-associated differential genes

To identify differentially expressed genes (DEGs) between PAAD and normal tissues, we employed the Limma package within the R software environment.^[17,18]^ Differences were deemed statistically significant when the false discovery rate was <0.05 and the absolute value of the log2 fold change (|log2(FC)|) was >1.0.^[19]^ Co-expression differential genes were then obtained from 3 datasets, TCGA, GSE28735, and GSE62452, using the online Wayne diagram (http://www.liuxiaoyuyuan.cn/) analysis website.^[20]^ Prognosis-related genes in PAAD were screened using the survival package in R. Subsequently, the obtained prognosis-related genes were imported into the TIMER database (https://cistrome.shinyapps.io/timer/) for further screening of immune-related prognosis genes.^[21,22]^

### 2.3. Prognostic assessment of target genes

The impacts of AHNAK2 expression and other clinical variables on OS were validated through analyses with the survival package in R. Samples that met the criterion of *P* < .1 in the univariate analysis were included in the multivariate Cox regression analysis to construct the model.^[23–25]^ Independent clinicopathological prognostic variables were selected through Cox regression analyses. Nomogram correlation models were then constructed and visualized using the Rms package in the R programming environment. These models were utilized to assess the OS rates at 1, 3, and 5 years in PAAD. Receiver operating characteristic (ROC) curves and time-dependent ROC curves were generated using the pROC package and the time ROC package in R, respectively. The results were subsequently visualized with the aid of the ggplot2 package.^[26,27]^

### 2.4. Functional enrichment analysis was performed on the target genes

To delve into the biological processes and pathways in which AHNAK2 might be implicated, we partitioned the PAAD samples into high and low expression groups. This was achieved by employing the median AHNAK2 expression level in the TCGA dataset as the cutoff value. Subsequently, the sequencing data of the 2 groups were differentially analyzed using the DESeq2 method to obtain AHNAK2-associated DEGs. The clusterProfiler package in R was then utilized to conduct GO, KEGG and Gene Set Enrichment Analysis (GSEA) on these AHNAK2-related differential genes.^[28–33]^ Additionally, the c2.cp.all.v2022.1.Hs.symbols.gmt subset was retrieved from the MSigDB database (https://www.gsea-msigdb.org/gsea/msigdb) to evaluate the associated pathways and molecular mechanisms.^[14,34]^ The obtained p-values were adjusted for multiple hypothesis testing using the Benjamini and Hochberg (BH) method. All the analyses were visualized with the R ggplot2 package.^[35]^

### 2.5. Analysis of the protein–protein interaction network of target genes

The STRING database (http://string-db.org) is a repository of protein interaction networks that aggregates information from public databases and the scientific literature. Meanwhile, Cytoscape (v3.9.1) is a software tool designed for visualizing molecular interaction networks and biological pathways. It also enables the integration of these networks with annotations, gene expression profiles, and other state-related data.^[28,36]^ A protein–protein interaction network (PPI) depicting the relationships between AHNAK2 and its associated genes was constructed. The underlying interaction data were sourced from the STRING database, which compiles a vast amount of information on protein-protein associations. Subsequently, the network was visualized and further analyzed using Cytoscape software, a well-recognized tool for representing and exploring complex biological interaction networks.

### 2.6. Analysis of immune infiltration associated with target genes

IOBR serves as a computational tool dedicated to immuno-oncology biology research. In this study, the immune infiltrating cell score for each sample was computed from our expression profiles. This calculation was carried out using the IOBR R package with the CIBERSORT method selected for the analysis.^[37,38]^ The association between AHNAK2 and each immune cell sub-population was ascertained using the Spearman correlation coefficient.

### 2.7. Analysis of target genes using the TISIDB database

The TISIDB database (http://cis.hku.hk/TISIDB/) is a comprehensive repository focused on tumor-immunity interactions. It has been established by integrating findings from research articles and various types of high-throughput data.^[39]^ We investigated the correlation between the expression levels of AHNAK2 and those of immune checkpoint genes, as well as chemokines/chemokine receptors, by utilizing the “Immunomodulators” and “Chemokines” modules of the TISIDB database.

### 2.8. Assessment of drug susceptibility

Gene expression data and drug activity-related data from the NCI-60 cell line panel were retrieved from the CellMiner database (https://discover.nci.nih.gov/cellminer). To conduct drug sensitivity analysis, we employed the “WGCNA,” “tidyr,” and “magrittr” packages in the R programming language. With a significance threshold of *P* < .05, we identified and presented the 4 drugs that showed the strongest correlations with the key expressed genes.^[40]^

### 2.9. Statistical analyses

Box plots were employed to evaluate the expression levels of the AHNAK2 gene in patients with PAAD. The data were presented as the median and interquartile range. The Wilcoxon rank-sum test was utilized to analyze the differences between the 2 groups. Kaplan—Meier survival curves were constructed using the survival and survminer packages in R and were statistically evaluated via the log-rank test. Moreover, hazard ratios along with 95% confidence intervals and log-rank P values were computed. Potential prognostic factors were assessed through univariate Cox regression analysis. The combined effect of AHNAK2 expression and other clinical variables on survival was confirmed by means of multivariate Cox regression analysis. All statistical analyses were carried out using R software (version 4.6).

## 3. Results

### 3.1. Identification of DEGs in PAAD

**Figure 2.**
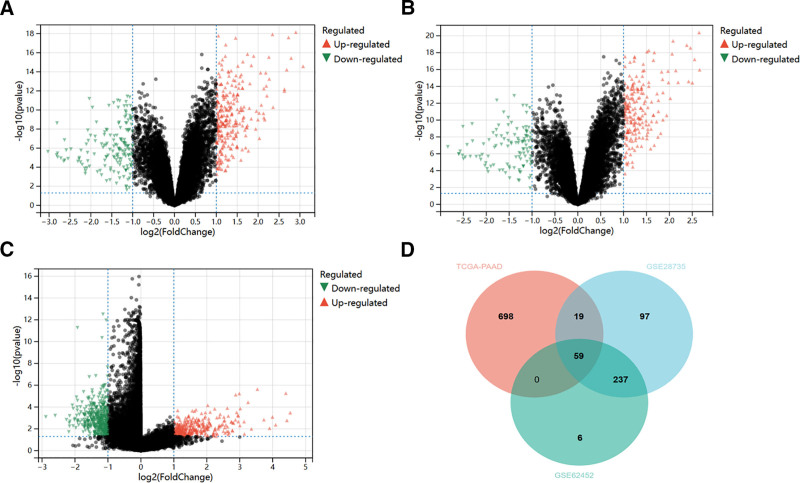
Screening of DEGs. (A) Volcano plot of DEGs in GSE28735 from the GEO database. (B) Volcano plot of DEGs in GSE62452 from the GEO database. (C) Volcano plot of DEGs in PAAD from TCGA database. (D) Intersection of DEGs from the GEO database and the TCGA database. (Image source: http://vip.sangerbox.com/home.html).

In the GSE28735 dataset, a total of 412 DEGs were identified through a rigorous screening process. Among them, 267 genes exhibited up-regulation, while 145 genes showed down-regulation. In the GSE62452 dataset, 302 DEGs were detected, with 208 being up-regulated and 94 down-regulated. Within TCGA dataset, 776 DEGs were found, comprising 320 up-regulated and 456 down-regulated genes. Subsequently, the DEGs from these 3 datasets were subjected to analysis using an online Venn diagram tool. This analysis led to the filtration of 59 co-expressed DEGs. Among these co-expressed genes, 58 were up-regulated, and only 1 was down -regulated (Fig. 2).

### 3.2. Identification of DEGs associated with immune prognosis

**Figure 3.**
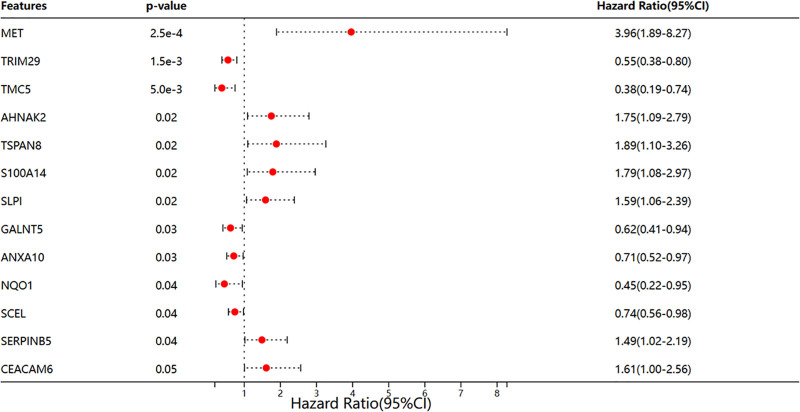
Forest plot depicting immune genes associated with prognosis. (Image source: http://vip.sangerbox.com/home.html).

**Figure 4.**
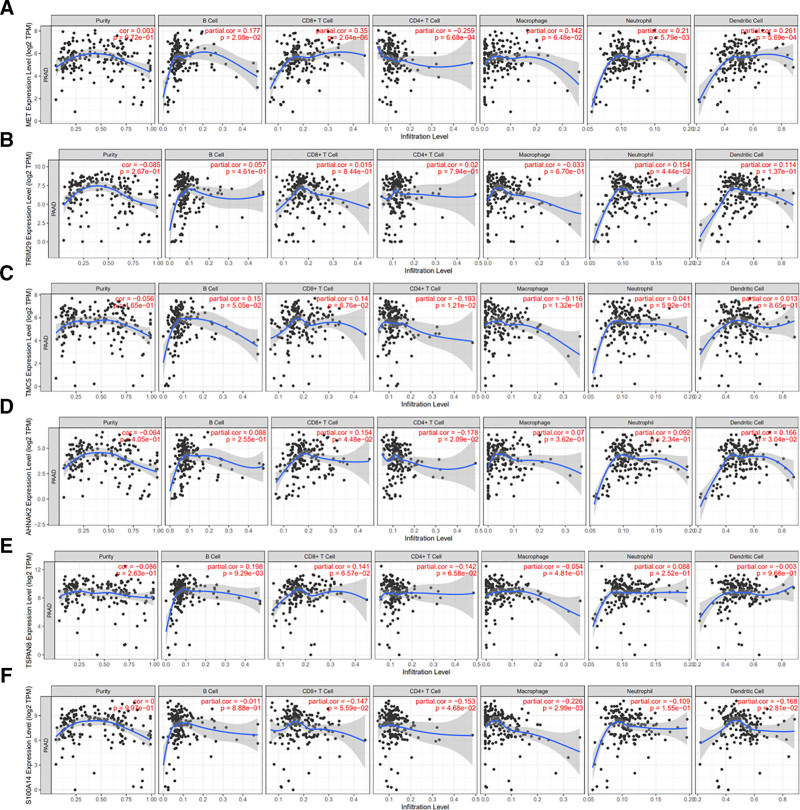
Correlation analysis was performed to explore the relationships between the expression level of AHNAK2 and the infiltration levels of B cells, CD8⁺ T cells, CD4⁺ T cells, macrophages, neutrophils, and dendritic cells. (Image source: https://cistrome.shinyapps.io/timer/).

Using the survival package in R software, we integrated data on the survival time and survival status of the samples. Subsequently, we employed the Cox proportional hazards model to evaluate the prognostic significance of DEGs. This analysis identified a total of 12 genes with prognostic relevance. Among these, MET, AHNAK2, TSPAN8, S100A14, SLPI, and SERPINB5 were associated with a higher risk of PAAD (Fig. 3). We then explored the correlation of these genes with 6 key immune cell types such as B cells, CD8⁺ T cells, CD4⁺ T cells, macrophages, neutrophils, and dendritic cells by using the TIMER database. The results indicated that the aforementioned 6 genes were immunologically relevant in the context of PAAD (Fig. 4). Notably, the function of AHNAK2 within the PAAD tumor microenvironment (TME) has been scarcely documented in the literature.

### 3.3. Differential expression analysis of AHNAK2

**Figure 5.**
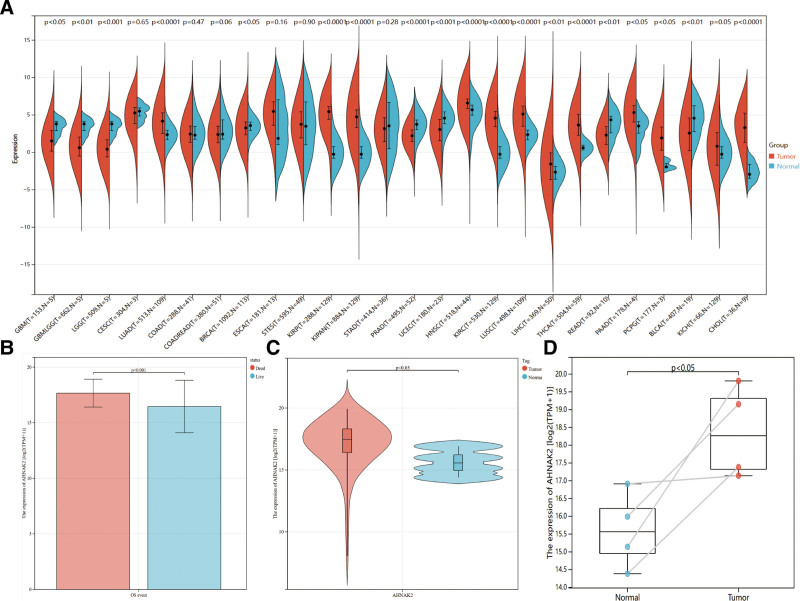
Differential expression analysis of AHNAK2. (A) AHNAK2 expression across diverse cancer types in the TCGA database. (B) Differential AHNAK2 expression in survival vs. death cases from the TCGA database. (C) Differential expression of AHNAK2 in unpaired samples from the TCGA database. (D) Differential expression of AHNAK2 in paired samples from the TCGA database. (Image source: http://vip.sangerbox.com/home.html).

To investigate the expression levels of AHNAK2 in normal tissues and tumors, we utilized the R software package to download and analyze AHNAK2 expression data from TCGA across various tumor types and their corresponding normal tissues. The analysis revealed that AHNAK2 was expressed at higher levels in several malignancies compared to normal tissues, including lung adenocarcinoma, kidney renal papillary cell carcinoma, head and neck squamous cell carcinoma, kidney renal clear cell carcinoma, and PAAD. Conversely, lower expression levels of AHNAK2 were observed in glioblastoma multiforme, glioma (GBMLGG), brain lower grade glioma, breast invasive carcinoma, and prostate adenocarcinoma (Fig. 5A).

The findings were further validated in PAAD tissues and their paired paracarcinoma tissues (Fig. 5C, D). Additionally, we examined the correlation between AHNAK2 expression and OS in PAAD patients. The analysis indicated a significant association between the expression level of AHNAK2 and OS (Fig. 5B).

### 3.4. AHNAK2 serves as an independent prognostic indicator for PAAD

Cox regression analysis was carried out to investigate the impacts of AHNAK2 expression and other clinical variables on OS. The univariate Cox regression model indicated that high AHNAK2 expression, T-stage, and N-stage were strongly linked to a poorer OS (*P* < .05; Table 1). Subsequently, these factors were incorporated into a multivariate Cox regression analysis. The results of this analysis demonstrated that AHNAK2 expression, T-stage, and N-stage were significantly associated with OS (*P* < .05; Table 1). Collectively, these data suggest that AHNAK2 expression can serve as an independent prognostic factor for OS in patients with PAAD.

**Table 1:** Univariate and multivariate Cox regression analysis of clinical characteristics.

Characteristics	n	Univariateanalysis		Multivariateanalysis	
		HR (95%Cl)	P	HR (95%Cl)	P
Gender	177				
Female	80	Reference			
Male	97	0.820 (0.54–1.24)	0.340		
Age (yr)	177				
≤65	93	Reference			
>65	84	1.31 (0.870–1.98)	0.200	1.13 (0.771–1.78)	0.488
Tstage	177				
T1 + T2	31	Reference			
T3 + T4	146	2.04 (1.080–3.85)	0.020	2.11 (1.22–4.23)	0.025
Nstage	177				
N0	49	Reference			
N1	128	2.11 (1.25–3.54)	0.004	2.015 (1.03–2.25)	0.0100
Stage	177				
Ⅰ+Ⅱ	167	Reference			
Ⅲ+Ⅳ	10	0.800 (0.250–2.53)	0.700	0.723 (0.325–1.13)	0.322
AHNAK2	177				
Low	68	Reference			
High	99	1.52 (1.33–1.83)	0.005	1.750 (1.09–2.79)	0.020

### 3.5. The diagnostic potential of AHNAK2 expression in PAAD

**Figure 6.**
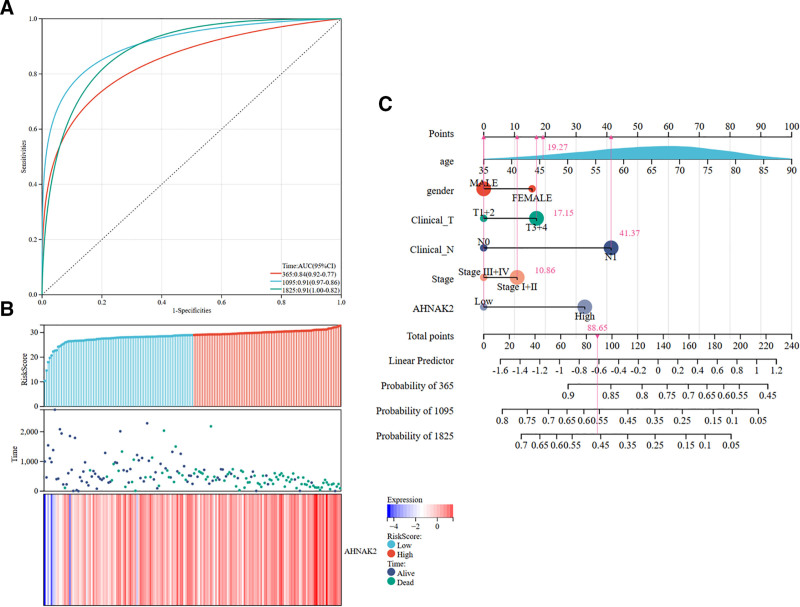
Diagnostic significance of AHNAK2 in PAAD. (A) Time-dependent ROC analysis of AHNAK2 expression. (B) Correlation between AHNAK2 expression and risk scores. (C) Prognostic columnar plot related to AHNAK2. (Image source: http://vip.sangerbox.com/home.html).

To assess the diagnostic potential of AHNAK2 expression in PAAD, we constructed a ROC curve and conducted a column-line graph analysis using AHNAK2 expression data fromTCGA database. The ROC curve is a graphical representation with the false-positive rate on the x-axis and the true-positive rate on the y-axis. The area under the ROC curve (AUC) is a widely used metric for evaluating the performance of diagnostic tests. An AUC value closer to 1 indicates a more effective diagnostic variable for predicting outcomes. In our study, the AUC values of AHNAK2 at 1, 3, and 5 years were 0.84, 0.91, and 0.91, respectively. All these values exceeded 0.50, suggesting that AHNAK2 has a high predictive accuracy (Fig. 6A). Simultaneously, we analyzed the relationship between AHNAK2 expression, patient follow-up time, and events. As the expression values of AHNAK2 increased (from left to right on the x-axis; refer to the top panel), there was a significant decline in patient survival (refer to the middle panel). As anticipated, the AHNAK2 gene acts as a risk factor, and its expression tends to be up-regulated with an increasing risk score (Fig. 6B).

Moreover, the results from the prognostic nomograms indicated that the predictive ability of the AHNAK2 expression level outperformed that of traditional clinical characteristics, including T-stage, N-stage, gender, overall stage, and age. Among these factors, the N-stage made the most substantial contribution to the prognosis (Fig. 6C).

### 3.6. Functional enrichment analysis of DEGs associated with AHNAK2 in PAAD

**Figure 7.**
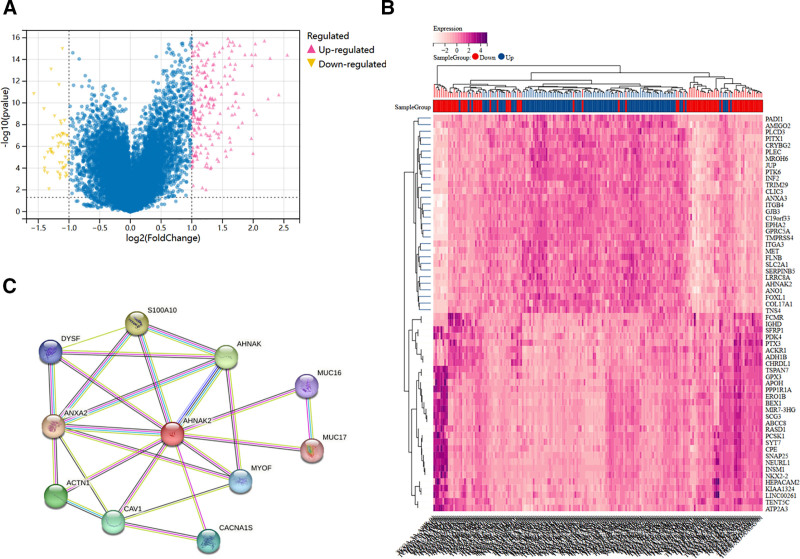
Screening of DEGs after re-grouping. (A) Volcano plot of DEGs. (B) Heat map of DEGs. (C) Analysis of protein–protein interaction network. (Image source: http://vip.sangerbox.com/home.html).

**Figure 8.**
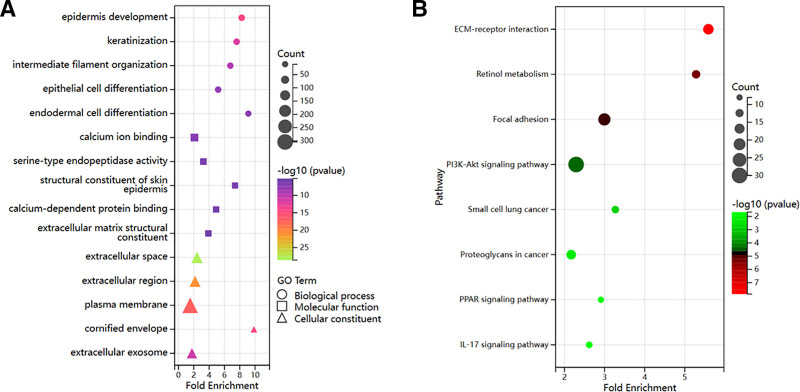
Functional enrichment analysis of AHNAK2-related DEGs. (A) Bubble plot for GO enrichment analysis of AHNAK2-related DEGs. (B) Bubble plot for KEGG enrichment analysis of AHNAK2-related DEGs. (Image source: http://vip.sangerbox.com/home.html).

To elucidate the biological function of AHNAK2 in PAAD, we partitioned the PAAD samples from TCGA into high and low-expression groups. The median expression level of AHNAK2 served as the cutoff value. Subsequently, we conducted separate analyses of the sequencing data from these 2 groups, generating volcano and heat maps to visualize the DEGs associated with AHNAK2 (Fig. 7A, B). Among the DEGs, 277 genes that met the criteria of |log₂(fold change)| > 1.5 and *P* < .05 were subjected to GO and KEGG enrichment analyses. The results indicated that these genes were predominantly enriched in processes such as epidermis development, keratinization, intermediate filament organization, and epithelial cell differentiation (Fig. 8A, B).

**Figure 9.**
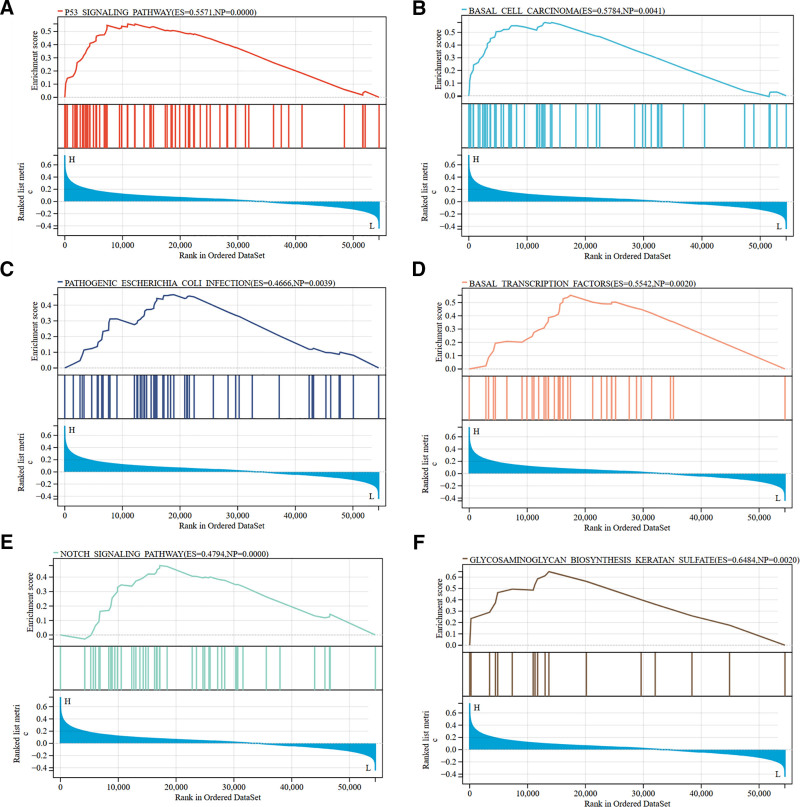
GSEA of DEGs. (A–F) Expansion of GSEA results for AHNAK2-related differential genes. (Image source: http://vip.sangerbox.com/home.html).

Moreover, we performed a GSEA on the DEGs associated with AHNAK2. The GSEA results revealed that high AHNAK2 expression was linked to multiple pathways. These included the p53 signaling pathway, basal cell carcinoma-related processes, pathogenic Escherichia coli infection, basal transcription factors, the Notch signaling pathway, and glycosaminoglycan biosynthesis-keratan sulfate, among others (Fig. 9A–F).

### 3.7. Analysis of PPI involving AHNAK2 and its associated genes

We searched for AHNAK2-associated genes on the STRING database and constructed a PPI network encompassing AHNAK2 and its interacting partners (Fig. 7C). The PPI analysis results demonstrated that AHNAK2 interacted with several key proteins, including actinin alpha 1 (ACTN1), AHNAK nucleoprotein (AHNAK), annexin A2 (ANXA2), calcium voltage-gated channel subunit alpha 1 S (CACNA1S), caveolin 1 (CAV1), dysferlin (DYSF), mucin 16 (MUC16), myoferlin (MYOF), S100 calcium-binding protein A10 (S100A10), and mucin 17 (MUC17). These interacting genes are predominantly involved in processes such as the positive regulation of plasma membrane repair, muscle cell development, muscle structure development, and the positive regulation of plasminogen activation. These findings suggest that AHNAK2 may play a crucial role in these fundamental biological pathways through its interactions with these associated proteins.

### 3.8. Correlation between AHNAK2 expression and immune characteristics

**Figure 10.**
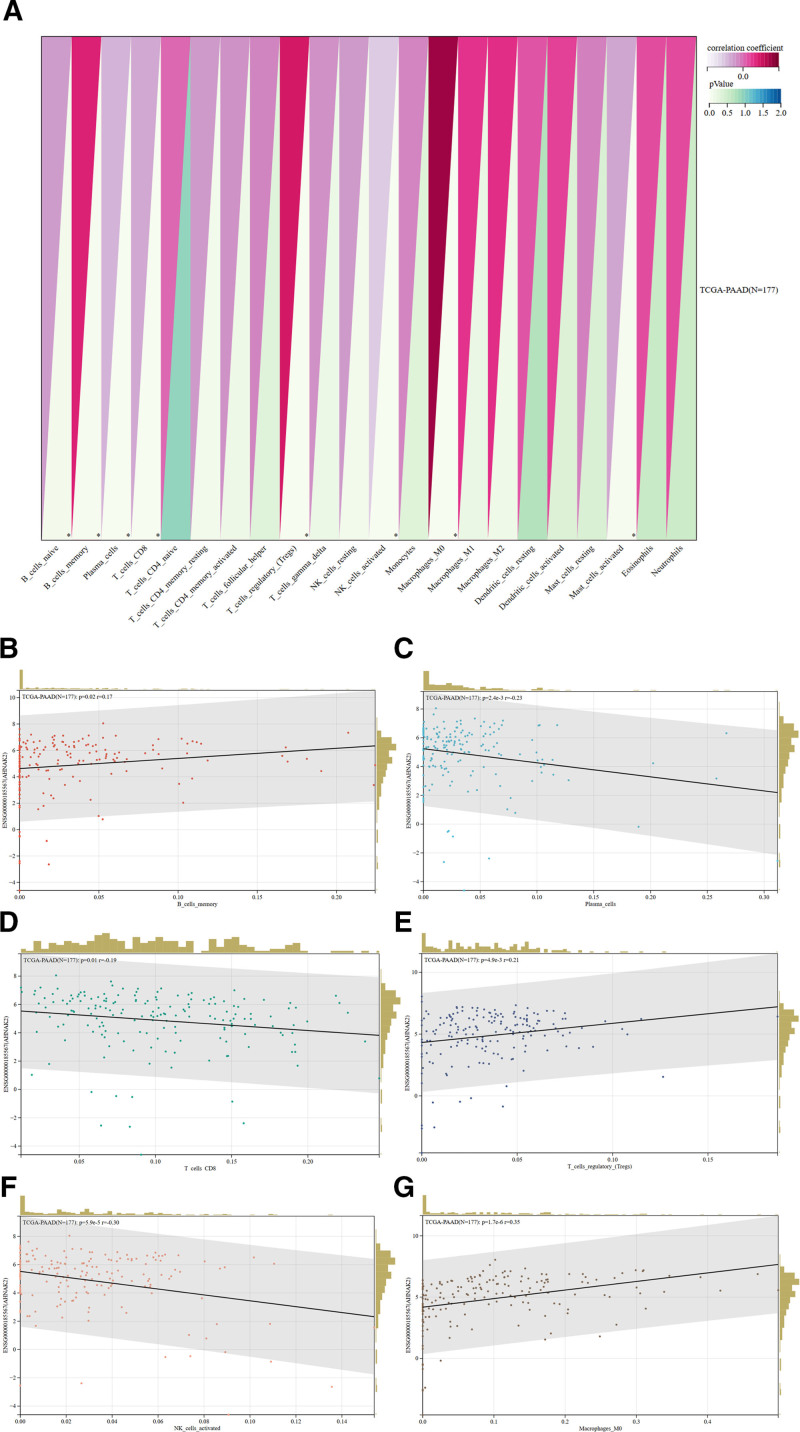
Immunoinfiltration analysis of AHNAK2. (A) Infiltration scores of AHNAK2 across 22 immune cell types. *P < .05, **P < .01. (B–G) Correlation between AHNAK2 expression and specific immune cell populations in the TCGA database. (Image source: http://vip.sangerbox.com/home.html).

Using the “corr.test” function from the “psych” package in R, we calculated the Pearson correlation coefficients between AHNAK2 expression and the infiltration scores of 22 types of immune cells to identify significantly correlated immune scores. Our analysis revealed a significant association between AHNAK2 and immune cell infiltration. Specifically, AHNAK2 showed positive correlations with memory B cells (*r* = 0.17, *P* < .05), regulatory T cells (*r* = 0.21, *P* < .01), and macrophages (*r* = 0.35, *P* < .001). In contrast, negative correlations were observed with plasma cells (*r* = −0.23, *P* < .01), CD8⁺ T cells (*r* = −0.19, *P* < .05), and natural killer (NK) cells (*r* = −0.30, *P* < .001) (Fig. 10A–G).

**Figure 11.**
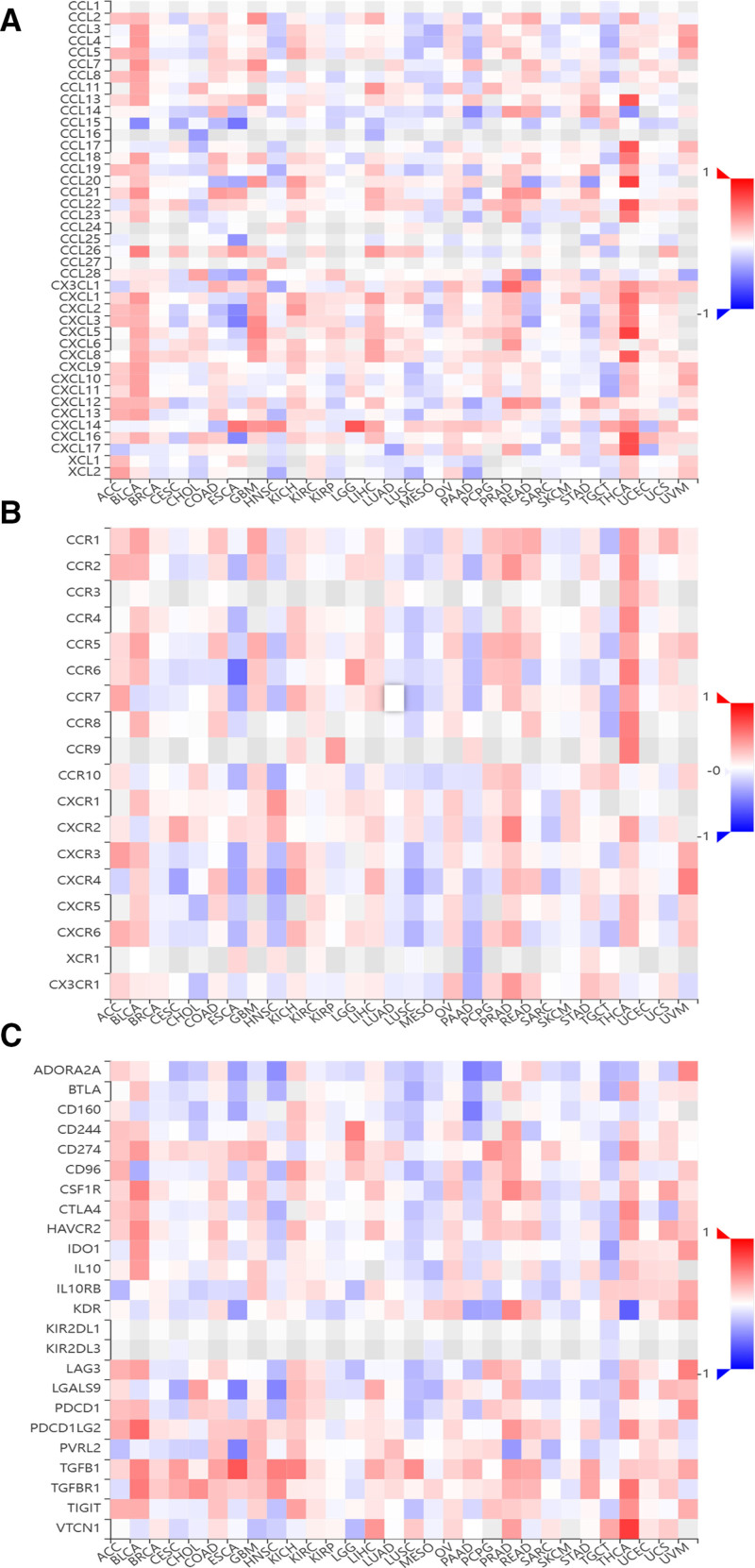
Heatmap-based analysis of the correlation between AHNAK2 and chemokines, chemokine receptors, and immunosuppressants in cancer. (A) Heatmap depicting the correlation between AHNAK2 and chemokines. (B) Heatmap depicting the correlation between AHNAK2 and chemokine receptors. (C) Heatmap depicting the correlation between AHNAK2 and immunosuppressants. (Image source: http://cis.hku.hk/TISIDB/).

**Figure 12.**
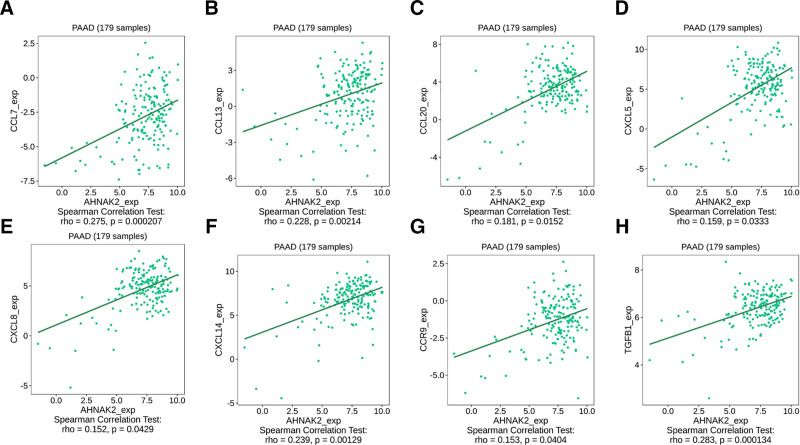
In PAAD, positive correlations between the expression of AHNAK2 and that of multiple molecules was identified. Specifically, these included CCL7 (as shown in panel a), CCL13 (panel b), CCL20 (panel c), CXCL5 (panel d), CXCL8 (panel e), CXCL14 (panel f), CCR9 (panel g), and TGFB1 (panel h). (Image source: http://cis.hku.hk/TISIDB/).

Moreover, we utilized the TISIDB database to analyze the correlation between AHNAK2 expression levels and the expression of immune cell-related chemokines and chemokine receptors in PAAD. The heatmap analysis demonstrated significant associations between AHNAK2 expression in PAAD and the expression of multiple chemokines and chemokine receptors. For instance, there were notable correlations with CCL7 (*r* = 0.275, *P *< .001), CCL13 (*r* = 0.228, *P* < .05), CCL20 (*r* = 0.181, *P *< .05), CXCL5 (*r* = 0.159, *P* < .05), CXCL8 (*r* = 0.152, *P* < .05), CXCL14 (*r* = 0.239, *P* < .05), and CCR9 (*r* = 0.153, *P* < .05) (Fig. 11A, B). Subsequently, we explored the relationship between AHNAK2 expression and the expression of immunomodulators across various human cancer types using the TISIDB database. The findings indicated a positive correlation between AHNAK2 expression and the expression level of TGFB1 (*r* = 0.283, *P* < .001) (Fig. 12). Collectively, these results imply that elevated AHNAK2 expression might be linked to an increased predisposition to cancer.

### 3.9. Drug sensitivity analysis

**Figure 13.**
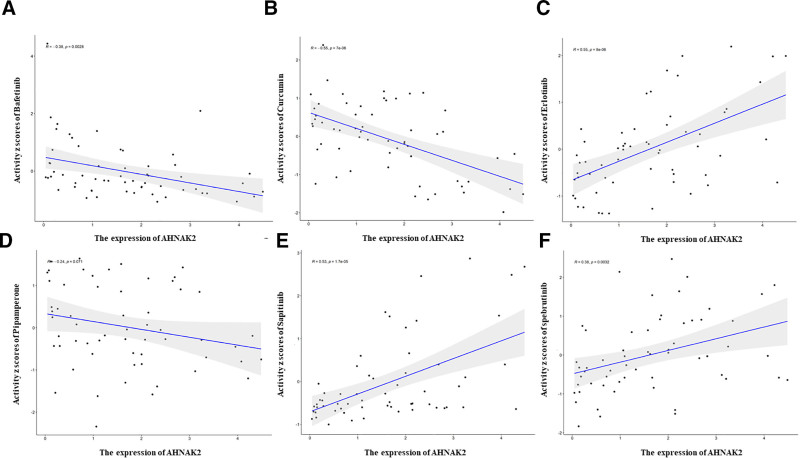
Drug sensitivity analysis of AHNAK2. (Image source: https://discover.nci.nih.gov/cellminer).

We investigated the expression levels of the key gene AHNAK2 and its correlation with chemotherapeutic agents in 60 human cancer cell lines from the NCI-60 panel. Previous research has suggested that AHNAK2 is associated with enhanced resistance to chemotherapeutic agents in various tumors. In our analysis, we found that AHNAK2 expression was strongly correlated with resistance to specific chemotherapeutic agents. Specifically, AHNAK2 expression demonstrated significant resistance to Bafetinib (*r* = −0.38, *P* < .05) and Curcumin (*r* = −0.55, *P* < .05) (Fig. 13).

## 4. Discussion

Pancreatic adenocarcinoma, often referred to as the “king of cancers,” is characterized by its high malignancy, low survival rate, insidious onset, challenges in early-stage detection, limited treatment options in advanced stages, and an extremely poor prognosis. This formidable disease poses a significant threat to human health and has remained a major focus of oncology research due to its complex biological behavior and dismal clinical outcomes.^[41,42]^ Immunotherapy has emerged as a prominent area of clinical research and has found applications in the treatment of multiple cancer types, including breast cancer,^[43]^ prostate cancer,^[44]^ liver cancer,^[45]^ lung cancer,^[46]^ and among others. Nonetheless, the targets of immunotherapy vary across different cancer types and patient populations, with distinct individualized differences emerging. The landscape of immunotherapy is far from uniform, as the biological characteristics of various cancers and the unique immune profiles of patients give rise to a spectrum of optimal targets, highlighting the need for personalized approaches in this rapidly evolving field of oncology.^[34,47]^ Therefore, immunological investigations focused on a specific cancer type hold the potential to unravel the underlying mechanisms of tumorigenesis and progression. Such studies can offer novel perspectives and valuable insights that may inform the development of more effective and targeted immunotherapeutic strategies for the future. This approach is crucial in the pursuit of personalized and precision oncology, aiming to optimize treatment outcomes and improve the prognosis of patients affected by the particular cancer under investigation.^[48]^

In the present study, through bioinformatics analysis, we identified AHNAK2 as a gene associated with the immune prognosis of PAAD.^[49]^ AHNAK2 plays a crucial role in several biological processes, including epidermal development, keratinization, intermediate filament organization, and epithelial cell differentiation. AHNAK2 belongs to the AHNAK family. Members of this family are implicated in the regulation of diverse biological functions, such as the modulation of calcium channels and membrane repair. Moreover, AHNAK2 has been shown to exert a regulatory influence on tumor progression, suggesting its potential as a key player in the pathophysiology of PAAD.^[50]^ Previous research^[51,52]^ has indicated that AHNAK2 participates in the autophagy process and modulates the proliferation, invasion, migration, and ferroptosis of lung adenocarcinoma cells. Moreover, prior research^[53]^ has demonstrated that AHNAK2 not only promotes the proliferation and confers drug resistance to pancreatic cancer cells via the activation of the KRAS/p53 pathway but also notably influences and cell-extracellular matrix adhesion. Additionally, it plays a crucial role in the regulation of the tumor immune microenvironment. Nevertheless, there is currently a dearth of reports regarding the immunization-related aspects of AHNAK2 in PAAD.

Here in this study, it was determined that the biological function and potential regulatory pathways of AHNAK2 in PAAD by integrating multiple bioinformatics analysis methods. First, we investigated the expression and prognostic value of the AHNAK2 gene in cancer. Our findings revealed a significant up-regulation of AHNAK2 expression in PAAD. In the context of PAAD, the expression level of AHNAK2 was strongly associated with OS, and it emerged as an independent prognostic factor for OS in PAAD patients. To uncover the potential regulatory pathways through which AHNAK2 influences PAAD prognosis, we conducted GO, KEGG, and GSEA on the DEGs related to AHNAK2. The results indicated that high AHNAK2 expression was linked to multiple pathways, including the p53 signaling pathway, processes associated with basal cell carcinoma, pathogenic Escherichia coli infection, basal transcription factors, the Notch signaling pathway, and glycosaminoglycan biosynthesis-keratan sulfate, among others.

Previous investigations^[54]^have demonstrated a strong association between the TME and the initiation and progression of cancer. Among them, immune cells account for a large portion of TME, and thus they play a key role in mediating pro- and anti-tumor immune responses.^[55]^ In our study, we observed a positive correlation between AHNAK2 expression and the infiltration levels of Tregs, memory B cells, and macrophages. Tregs, a distinct subset of T cells that make up 5% to 10% of the total CD4⁺ T cell population, play a crucial role in maintaining immune homeostasis and preventing autoimmunity. Their presence and activity within the TME can have significant implications for the immune response against cancer and the overall progression of the disease. The observed association between AHNAK2 and these immune cell types may provide new insights into the immunomodulatory mechanisms of AHNAK2 in the context of cancer.^[56]^ Tregs can secrete inhibitory cytokines, including IL-10, TGF-β, and IL-35, and can also kill effector cells through granzyme and perforin.^[57,58]^ It has also been reported^[59]^ that Tregs compete with effector T cells to deplete IL-2, thereby inhibiting the growth of effector T cells. In addition, Tregs transfer large amounts of cAMP to effector T cells through gap junctions, interfering with their metabolism.^[60]^ Cysneiros et al.^[61]^demonstrated the involvement of Tregs in the PAAD immune escape resistance process. Thus, it appears that AHNAK2 may play an important role in TME remodeling as well as in the development and metastasis of PAAD and is associated with a poor prognosis of PAAD.

Chemokines, which belong to a subfamily of cytokines, play a pivotal role in regulating immune cell trafficking and the development of lymphoid tissues. Through these functions, they exert a profound influence on tumor progression, treatment efficacy, and patient prognosis by molding the immune and biological phenotypes of tumors.^[62,63]^ In the present study, we discovered a significant positive correlation between the expression level of AHNAK2 and several chemokines/chemokine receptors, including CCL7, CCL13, CXCL20, CXCL5, and CCR9. Several investigations^[64,65]^ have reported a strong association between CCL7, CCL13, CXCL5, and CCR9 and tumorigenesis as well as tumor development. Furthermore, previous research^[66]^ has indicated that the interaction between CCR9 and CCL25 may have a pivotal role in facilitating the metastasis of plasmacytoid dendritic cells (PDCs) to metastatic lymph nodes and cancerous tissues. Moreover, CCR9 has been shown to influence cell migration towards inflammatory tissues by modulating the expression of T regulatory (Treg) cells. This regulatory mechanism leads to a cascade of multidimensional immune responses and disrupts immune homeostasis.^[67]^ The above statement may explain the mechanism by which AHNAK2 regulates the immune infiltration of PAAD. Furthermore, in PAAD, we observed a positive correlation between AHNAK2 and the expression level of TGFB1, an immunonegative regulator. This finding further implies that elevated AHNAK2 expression might be intricately linked to cancer development. The interaction between AHNAK2 and TGFB1 could potentially contribute to the establishment of an immunosuppressive microenvironment, facilitating tumor progression and highlighting AHNAK2 as a potential target for cancer-related research and therapeutic interventions.

Drug resistance is a difficult point that cannot be bypassed in cancer treatment. Even if good results are obtained in the preliminary treatment, resistance to therapeutic drugs eventually arises because of differences in the time and dose of administration.^[68–71]^ In recent years, resistance to chemotherapeutic drugs for pancreatic cancer has been successively reported, and gene therapy is expected to become a novel treatment method.^[72,73]^ We investigated the expression level of AHNAK2 in 60 human cancer cell lines (NCI-60) and its correlation with chemotherapeutic agents and found that AHNAK2 was associated with increased resistance to chemotherapeutic agents in a variety of tumors. The expression of AHNAK2 showed strong resistance to Bafetinib and Curcumin.

Despite conducting a comprehensive analysis of AHNAK2 and performing cross-validation using diverse databases, direct evidence establishing that AHNAK2 impacts patient prognosis through its involvement in immune infiltration remains lacking. Future experimental investigations are thus warranted to further validate the functions of the AHNAK2 gene.

In summary, our study revealed a significant up-regulation of AHNAK2 expression in PAAD. The expression level of AHNAK2 holds diagnostic and prognostic value for PAAD. Mechanistically, AHNAK2 may influence the progression of PAAD by reshaping the TME. These findings suggest that AHNAK2 has the potential to serve as a valuable biomolecular marker for immunotherapy in patients with PAAD, paving the way for more targeted and effective treatment strategies in the future.

However, this study still has several limitations. First, the limited number of chip datasets included restricted the breadth of the research; moreover, differences in data processing methods across databases may have affected the accuracy of the results. Second, tumor immune infiltration is jointly regulated by multiple inflammatory factors to maintain homeostasis within the microenvironment. This study focused solely on immune cells currently in the research spotlight, potentially overlooking the regulatory roles of other immune cells. Finally, while some literature supports the plausibility of our conclusions, this study did not conduct experimental validation of the results. Given these limitations, the research team will initiate experimental designs to validate the key genes/immune biomarkers identified in this study. This will involve cell functional assays (e.g., proliferation and apoptosis assays) and animal model validation (e.g., drug response experiments in xenograft models). The corresponding results will be published in subsequent studies.

## Acknowledgments

We extend our sincere gratitude to Qinyan Wu for his invaluable contributions to this work, despite not being included among the authors. His assistance has been instrumental in the successful execution of this study, and his expertise and insights have significantly enhanced the quality and depth of our research.

## Author contributions

**Methodology:** Bo Wu, Yixin Liu.

**Writing – original draft:** Bo Wu.

**Conceptualization:** Yixin Liu.

**Formal analysis:** Xiaohong Lan.

**Software:** Xiaohong Lan, Yang Yang, Xuqing Chen.

**Supervision:** Yuekun Wang.

**Validation:** Yuekun Wang, Yang Yang.

**Visualization:** Yuekun Wang, Yang Yang.

**Writing – review & editing:** Xuqing Chen.
